# The Thioredoxin Fold Protein (TFP2) from Extreme Acidophilic *Leptospirillum* sp. CF-1 Is a Chaperedoxin-like Protein That Prevents the Aggregation of Proteins under Oxidative Stress

**DOI:** 10.3390/ijms25136905

**Published:** 2024-06-24

**Authors:** Claudia Muñoz-Villagrán, Javiera Acevedo-Arbunic, Elisabeth Härtig, Susanne Sievers, Daniela Zühlke, Francisco Issotta, Carolina Mascayano, Dieter Jahn, Martina Jahn, Gloria Levicán

**Affiliations:** 1 Laboratorio de Microbiología Básica y Aplicada, Departamento de Biología, Facultad de Química y Biología, Universidad de Santiago de Chile (USACH), Santiago 9170022, Chile; 2 Institute of Microbiology, Technische Universität Braunschweig, Spielmannstr 7, 38106 Braunschweig, Germany; e.haertig@tu-braunschweig.de (E.H.); 3 Department of Microbial Physiology and Molecular Biology, Institute of Microbiology, University of Greifswald, 17489 Greifswald, Germany; daniela.zuehlke@uni-greifswald.de (D.Z.); 4 Departamento Genética Molecular y Microbiología, Facultad de Ciencias Biológicas, Pontificia Universidad Católica, Santiago 8331150, Chile; 5 Laboratorio de Simulación Computacional y Diseño Racional de Fármacos, Departamento de Ciencias del Ambiente, Facultad de Química y Biología, Universidad de Santiago de Chile (USACH), Santiago 9170022, Chile; 6 Braunschweig Integrated Centre of Systems Biology BRICS, Technische Universität Braunschweig, Rebenring 56, 38106 Braunschweig, Germany

**Keywords:** acidophiles, chaperedoxin, CnoX, *Leptospirillum*, TFP2, thioredoxin, protein aggregation, proteostasis

## Abstract

Extreme acidophilic bacteria like *Leptospirillum* sp. require an efficient enzyme system to counteract strong oxygen stress conditions in their natural habitat. The genome of *Leptospirillum* sp. CF-1 encodes the thioredoxin-fold protein TFP2, which exhibits a high structural similarity to the thioredoxin domain of *E. coli* CnoX. CnoX from *Escherichia coli* is a chaperedoxin that protects protein substrates from oxidative stress conditions using its holdase function and a subsequent transfer to foldase chaperones for refolding. Recombinantly produced and purified *Leptospirillum* sp. TFP2 possesses both thioredoxin and chaperone holdase activities in vitro. It can be reduced by thioredoxin reductase (TrxR). The *tfp2* gene co-locates with genes for the chaperone foldase GroES/EL on the chromosome. The “*tfp2* cluster” (*ctpA-groES-groEL-hyp-tfp2-recN*) was found between 1.9 and 8.8-fold transcriptionally up-regulated in response to 1 mM hydrogen peroxide (H_2_O_2_). *Leptospirillum* sp. *tfp2* heterologously expressed in *E. coli* wild type and *cnoX* mutant strains lead to an increased tolerance of these *E. coli* strains to H_2_O_2_ and significantly reduced intracellular protein aggregates. Finally, a proteomic analysis of protein aggregates produced in *E. coli* upon exposition to oxidative stress with 4 mM H_2_O_2_, showed that *Leptospirillum* sp. *tfp2* expression caused a significant decrease in the aggregation of 124 proteins belonging to fifteen different metabolic categories. These included several known substrates of DnaK and GroEL/ES. These findings demonstrate that *Leptospirillum* sp. TFP2 is a chaperedoxin-like protein, acting as a key player in the control of cellular proteostasis under highly oxidative conditions that prevail in extreme acidic environments.

## 1. Introduction

In bacteria, the intracellular accumulation of reactive oxygen species (ROS) can occur as a response to a wide range of exogenous agents, such as excess UV radiation, metal ions, and osmotic stress, among other factors [1]. This phenomenon can cause oxidative stress, which can lead to the generalized oxidation of biomolecules [1]. While the exact percentage varies depending on the specific bacterial species and growth conditions, proteins typically constitute a substantial fraction of the biomolecular content in bacterial cells. In this way, they are thus susceptible to denaturation, unfolding, and aggregation in the cytoplasm under oxidative conditions [2]. In addition, an increased level of ROS can down-regulate global protein synthesis [3]. Therefore, under oxidative stress conditions, the coordinated action of redox-responsive chaperones is an essential component of the molecular response that protects proteins and safeguards homeostasis of the cellular proteome.

In *Escherichia coli*, protection of the proteome against irreversible damage involves the action of molecular chaperones that prevent aggregation, and proteases that degrade proteins to amino acids for reuse [4]. In bacteria, disaggregation and folding are mediated by ATP-dependent foldase chaperones such as DnaK (Hsp70), as well as GroEL (Hsp60), HtpG (Hsp90), and ClpB (Hsp104) [5,6]. During oxidative stress, the accumulation of ROS can alter the activity of foldases due to one or a combination of the following effects: (i) the damage is extremely rapid and affects a wide range of proteins simultaneously, including chaperones; (ii) the direct sensitivity of some chaperones to oxidative inactivation (e.g., GroEL by hypochlorous acid and peroxynitrite) [7]; and (iii) a decrease in ATP levels modifies the activity of at least some of the classical ATP-dependent foldases—for instance, DnaK is inactivated by a decrease in intracellular ATP levels [8]. Consequently, cells make use of different strategies to specifically deal with oxidative protein damage. One of these is the activation of ATP-independent chaperone holdases that are redox-regulated, such as Hsp33 and CnoX [9]. In contrast to foldases like DnaK/J and GroEL/ES that actively promote folding, holdases bind unfolded proteins in an ATP-independent manner to prevent their aggregation [9].

CnoX (formerly YbbN) is a relatively recently discovered type of chaperone described in *E. coli* [10]. This protein combines holdase activity with the ability to protect its substrates from irreversible oxidation, thus avoiding aggregation and overoxidation of the client proteins. Given this dual function, CnoX has been ascribed to a new category of proteins known as chaperedoxins [10]. CnoX consists of a thioredoxin domain (Trx) and a tetratricopeptide-repeat (TRP) domain [11]. The thioredoxin domain catalyzes the reduction of oxidized cysteine residues in proteins [12], whilst TRP motifs usually mediate protein–protein interactions [13]. CnoX binds to unfolded proteins and, after redox homeostasis is re-established, transfers its substrates to DnaK/J and GroEL/ES for refolding [10]. In order to remain active, the Trx domain of CnoX is reduced by thioredoxin reductase (TrxR) [12].

Highly oxidative environmental conditions are endured by many bacterial species, implying a requirement for similar mechanisms to maintain cellular proteostasis. One such species is *Leptospirillum* sp., an iron-oxidizing acidophilic bacteria from the Nitrospiria class (phylum Nistrospirota) that is abundant in microbial communities involved in bioleaching of metals from sulfide minerals [14]. In natural environments or industrial operations for metal recovery, these microorganisms are exposed to high-concentration metals such as copper, iron, and zinc, among others [15]. *Leptospirillum* sp. and other acidophiles also face ROS that have been spontaneously generated and which accumulate on the surface of sulfide minerals like pyrite (FeS_2_), chalcopyrite (CuFeS_2_), and spharelite (ZnS) [16,17,18]. Additionally, factors such as extremely acidic pH and high osmolarity are potential contributors to the internal generation of ROS in the cell [19]. Thus, it is envisioned that these bacteria must possess efficient mechanisms of response that protect the proteome and facilitate cell proteostasis under redox variations. However, the understanding of the molecular mechanisms of proteostasis in *Leptospirillum* and other acidophiles is still limited.

In *Leptospirillum* sp. CF-1, a total of 13 *tfp* genes that encode thioredoxin fold proteins have been described [20]. These thirteen genes are all transcriptionally active and are predicted to be involved in several cellular functions. Some of these genes confer oxidative protection to a thioredoxin-deficient *E. coli* strain by restoring wild-type characteristics when heterologously expressed [21]. Notably, the presence of the gene *tfp2* co-localizes and coexpresses with genes for GroES and GroEL chaperones. Interestingly, the TFP2 protein has a similarity with the N-terminal amino acid residues of the chaperedoxin CnoX from *E. coli* [21]. Therefore, in this work, we have addressed the study of the thioredoxin fold protein TFP2 of *Leptospirillum* sp. CF-1 in order to evaluate its role as a chaperedoxin-like protein that acts by preventing the aggregation of proteins under conditions of oxidative stress.

## 2. Results

### 2.1. Structural and Multiple Sequence Alignment Analysis of tfp2 from Leptospirillum sp. CF-1

**Figure 1:**
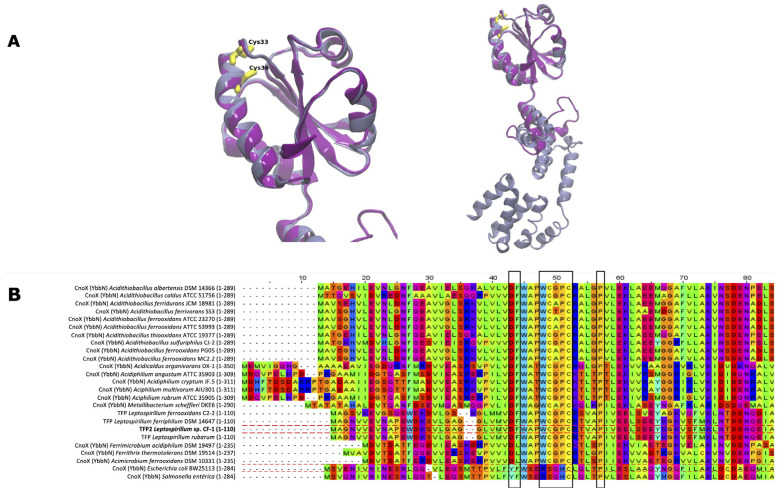
Structural and multiple sequence alignment of TFP2. (**A**) Model of TFP2 from *Leptospirillum* sp. CF-1 (purple) and CnoX from *E. coli* (gray) obtained using Modeller. The boxes highlight residues with key structural and functional roles: Asp27 and Phe28 (DF), Pro35-41 (P), and Cys33 and Cys36 from the CXXC motif. (**B**) Multiple sequence alignment of thioredoxin in selected acidophilic and neutrophilic bacteria.

The *tfp2* gene encodes a predicted protein of 110 amino acids. As mentioned above, the gene co-localizes with genes for chaperones GroES and GroEL, and showed similarity (55%) with the N-terminal amino acid residues of CnoX (residues 1–95), a chaperedoxin from *E. coli*. Based on these findings, we conducted a structure alignment between TFP2 and CnoX_Ec. The model of TFP2 was obtained by Modeller and then assessed and validated as described in Material and Methods. TFP2 showed a high correspondence with the thioredoxin domain of CnoX from *E. coli*, with the canonical presence of five beta strands surrounded by four alpha helices (Figure 1A). However, it was not possible to detect the tetratricopeptide repeat (TPR) domain described in CnoX_Ec (Figure 1A).

The Trx domain that is present in the thioredoxin family of proteins possesses a characteristic CXXC motif in the active site (some canonical thioredoxins possess a WCGPC active site) around residue 30 [22]. It is possible to observe the conservation of both Cys33 and Cys36, which could participate in chaperedoxin activity. Interestingly, the comparison of CnoX sequences of several acidophiles with TFP2 of the *Leptospirillum* genus (Figure 1B) showed that all possess the conserved sequence WCXXC, contrary to what was observed in neutrophils that harbor the CXXC (*Metallibacterium scheffleri*) or SXXC motifs (*E. coli* and *Salmonella enterica*) (second black frame).

### 2.2. Evaluation of Reductase and Chaperone Activities of TFP2 In Vitro

**Figure 2:**
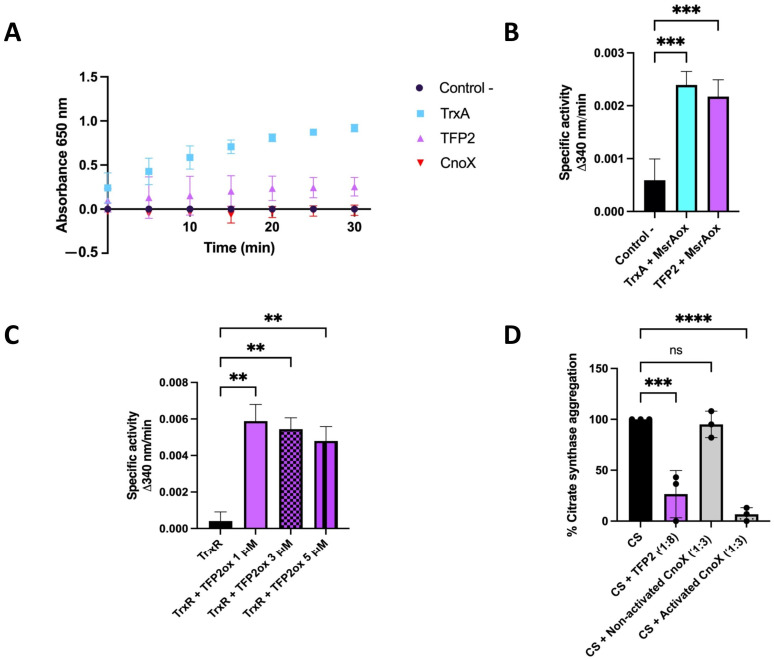
Determination of reductase activities of purified TFP2 from *Leptospirillum* sp. CF-1. (**A**) Thioredoxin activity based on the reduction of insulin disulfide by DTT; (**B**) thiol reductase activity based on NADPH consumption by oxidized MsrA; (**C**) reduction of TFP2 by TrxR based on NADPH consumption; (**D**) chaperone activity determined by the aggregation of citrate synthase (CS) at 43 °C. Activation of CnoX was mediated by prior incubation with 2 mM HOCl for 10 min at room temperature. (**) *p* < 0.01; (***) *p* < 0.001; (****) *p* < 0.0001; (ns) not significant.

In order to assess whether TFP2 exhibits classical thioredoxin activity, we first conducted tests to determine whether purified recombinant TFP2 (his-tagged) is able to reduce insulin [23]. As shown in Figure 2A, thioredoxin activity of TFP2 was detected in all measurements performed during a period of 30 min. The activity of TFP2 was significantly lower than that of thioredoxin 1 from *E. coli* (TrxA_Ec), although it was markedly higher than that of CnoX_Ec. The thiol reductase activity of TFP2 was also evaluated on oxidized MsrA enzyme, a classical thioredoxin substrate, and measured by the consumption of NADPH over time. As can be observed in Figure 2B, TFP2 exhibited oxidoreductase activity that was in the same range as that of TrxA_Ec. Finally, we investigated whether TFP2 could be reduced, and thus recovered, by the NADPH-dependent flavoenzyme thioredoxin reductase (TrxR) by measuring NADPH consumption. Our observations indicate that TFP2 oxidized by diamide could be efficiently recycled by TrxR (Figure 2C).

To determine holdase chaperone activity, we employed an assay based on the thermolabile enzyme citrate synthase (CS), which had been unfolded by incubation at 43 °C. The aggregation of CS was assessed using light scattering at 360 nm [10]. The results demonstrate that the presence of TFP2 during this incubation significantly prevented the aggregation of CS, strongly suggesting that this protein does indeed possess holdase activity (Figure 2D). Interestingly, and similar to that described for CnoX from *Caulobacter crescentus*, the holdase activity of TFP2 did not require prior activation by HOCl, as is the case for CnoX_Ec [24].

### 2.3. Role of TFP2 in Protecting E. coli against Oxidative Stress

**Figure 3:**
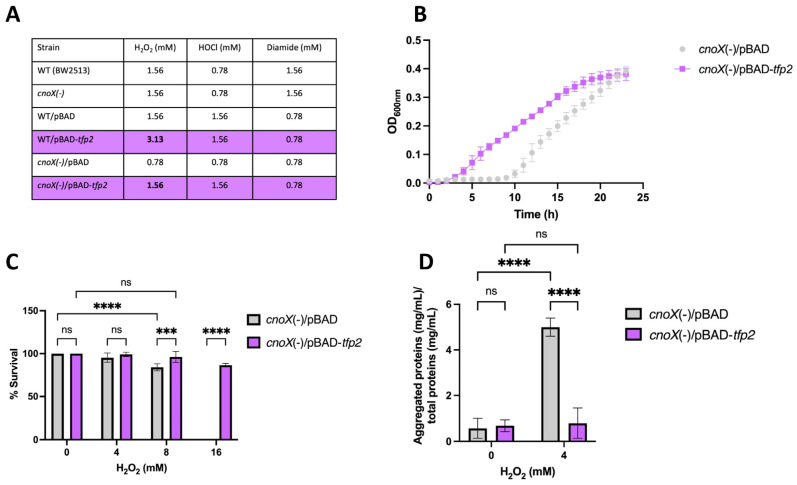
Effect of *tfp2* on the tolerance to oxidative conditions of *E. coli.* (**A**) MIC for H_2_O_2_, HOCl and diamide. (**B**) Bacterial growth in the presence of 0.39 mM H_2_O_2_ (1/2 MIC). (**C**) Viability and (**D**) Content of protein aggregates in cells grown under oxidative conditions for 2 h. (***) *p* < 0.001; (****) *p* < 0.0001; (ns) not significant.

To determine the ability of *E. coli* strains complemented with *tfp2* to withstand oxidative stress, the gene was cloned into the pBAD TOPO vector, which was then transformed into *E. coli* WT (BW25113) and *cnoX*(-). Resistance to H_2_O_2_, diamide, and HOCl was assessed by determining the corresponding minimal inhibitory concentration (MIC). The results (Figure 3A) reveal that both the WT and the *cnoX* mutant strains complemented with the pBAD-*tfp2* vector exhibited an increase in resistance to H_2_O_2_ (doubling the MIC) compared to the empty vector. However, in agreement with the results described above, the same effect was not observed in wild-type cells treated with HOCl and diamide.

On the other hand, in order to evaluate susceptibility to H_2_O_2_, we measured the growth and viability of the *E. coli cnoX* mutant exposed to 0.39 mM H_2_O_2_ (1/2 MIC). As shown in Figure 3B, exposure of the *cnoX* mutant harboring the empty vector to peroxide resulted in a lag phase that extended for 10 h; however, cell cultures complemented with the vector carrying *tfp2* showed a considerably shorter lag phase of only 2 h. In addition, the cultures showed significant differences in percentages of survival after exposure for 2 h to 8 and 16 mM H_2_O_2_ (Figure 3C). Of note is that exposure to 16 mM H_2_O_2_ led to a total loss of survival of *E. coli*, while the presence and expression of *tfp2* allowed the almost complete restoration of this parameter (86.6%). These results clearly show a positive effect of *tfp2* in enhancing the tolerance of *E. coli* to H_2_O_2_. The same effects were not observed for HOCl and diamide exposure.

Finally, we assessed the formation of protein aggregates in cells exposed to 4 mM H_2_O_2_. After stress induction, protein aggregates were collected and quantified as described in Materials and Methods. The results indicate that the presence and expression of *tfp2* generated a significant decrease (by 6.3 fold) in aggregated protein content compared to cells that harbored the empty vector (Figure 3D, reinforcing the idea that TFP2 possesses holdase activity that contributes to diminishing protein coagulation under oxidative stress conditions without the requirement of HOCl activation.

### 2.4. Genetic Organization and Expression of tfp2 Gene and Neighboring Genes

**Figure 4:**
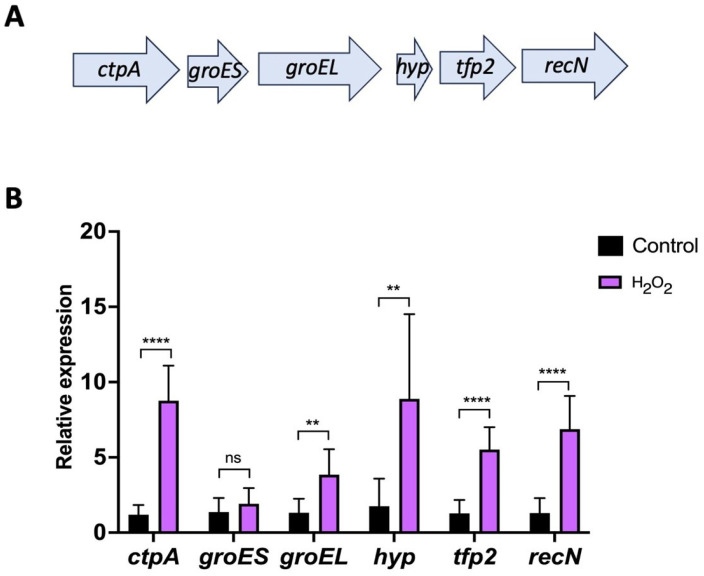
Expression of the “*tfp2* cluster” from *Leptospirillum* sp. CF-1. (**A**) Genetic context surrounding *tfp2*. Genes of the cluster encode the C-terminal protease CtpA (*ctpA*), the chaperone foldase system GroEL/ES (*groES*, *groEL*), the hypothetical protein Hyp (*hyp*), the thioredoxin-fold protein TFP2 (*tfp2*), and the recombination and DNA repair protein RecN (*recN*). (**B**) Effect of H_2_O_2_ exposure on the relative expression of *tfp2* and neighboring genes. Data represent the average of three independent experiments. Statistical analysis was carried out using the Student-t test. (**) *p* < 0.01; (****) *p* < 0.0001; (ns) not significant.

In a previous study, we demonstrated that the expression of *tfp2* in *Leptospirillum* sp. CF-1 was up-regulated in response to diamide and ferric iron [21]. Herein, we were interested in analyzing the expression pattern of *tfp2* and the neighboring genes upon exposure to peroxide. The analysis of the genetic neighborhood upstream of *tfp2* showed the presence of genes encoding the carboxyl-terminal processing protease CtpA, the chaperone complex GroEL/ES, and a hypothetical protein (Hyp). Downstream, the gene encoding for the DNA repair protein RecN was found (Figure 4A). Thus, the entire locus contained genes that encode proteins related to protein homeostasis and DNA protection. Interestingly, the *hyp* gene adjacent to *tfp2* encoded a small hypothetical protein (47 amino acids) that has similarity (30%) in a segment of 60 amino acids with the TRP domain present in CnoX_Ec. Whether this TRP small-protein favors the annealing and interaction of the mis- or un-folded proteins with TFP2 [6] should be evaluated. 

To evaluate the role of the “*tfp2* cluster” in oxidative defense, the mRNA levels of each of the six genes present in this locus were evaluated using RT-qPCR assays when *Leptospirillum* sp. CF-1 was exposed to 1 mM H_2_O_2_. The results show an increase in the level of transcripts of all genes (*ctpA*: 8.8, *groES*: 1.9; *groEL*: 3.8, *hyp*: 8.9, *tfp2*: 5.5, and *recN*: 6.9 folds change) (Figure 4B) suggesting an active role of the proteins encoded against redox stress. The genetic organization of the “*tfp2* cluster” and the expression patterns of the genes under prooxidant conditions agree with a possible role of TFP2-like chaperedoxin, which could cooperate with the chaperone GroEL/ES to protect and fold mis- or un-folded proteins. In addition, it is tempting to postulate a possible role of protease CtpA in the degradation of unfolded proteins that cannot be rescued by the chaperone machinery of the cell.

### 2.5. Identification of TFP2 Client Proteins

To date, there is no genetic system that allows the generation of mutants or the overexpression of genes in *Leptospirillum* CF-1; therefore, it is not possible to identify potential client proteins by direct analysis in the strain CF-1. However, the results above (Figure 3D) indicate that the overexpression of TFP2 in the CF-1 strain of *E. coli* (*tfp2*_Ec) significantly decreases the aggregated protein content relative to cells that carry the empty vector (ev_Ec) when both are exposed to 4 mM H_2_O_2_. These results suggest the existence of a protein set from *E. coli* that could be substrates of TFP2. In order to identify potential TFP2 client proteins, we conducted a proteomic study of aggregated proteins in response to peroxide treatment in ev_Ec and *tfp2*_Ec cells, using LC-MS/MS analysis (Experimental design in Figure S1). Total proteomic data can be found in the Supplementary Materials (Table S3). We identified 1316 proteins in the aggregates from ev_Ec-cells and 1560 from *tfp2*_Ec-cells (in at least one of three replicas). In both cell types, we identified 38 (Table S1) and 150 (Table S2) unique proteins, respectively. This comparison allowed us to identify 124 potential TFP2’s client proteins (downregulated in protein aggregates from *tfp2*_Ec cells) that were related to fifteen metabolic categories, including energy conversion, transport, and metabolism of amino acids/nucleotides/carbohydrates/coenzymes/lipids, translation, transcription, posttranslational modification/protein turnover/chaperones, and defense mechanisms (Table 1). Furthermore, a bioinformatic analysis of these proteins to evaluate their propensity to aggregate and their cysteine content enabled us to establish that 80% possess between 0.18–4.26% of cysteine content (2–9 residues), and that the vast majority have a low propensity to aggregate (Table S1). These findings suggest that their aggregation is mainly due to the stress conditions applied to the cells and that the great majority are substrates of chaperedoxin activity. In addition, 119 of the 124 listed proteins were predicted as potential substrates of DnaK and/or GroEL/ES. Therefore, as described for CnoX_Ec substrates [11,25], TFP2 from the strain CF-1 could play a potential role in protecting and maintaining a fold-competent state of a diversity of proteins that participate in multiple cellular processes, thus playing a crucial role in cell physiology and adaptation. It should be noted that 70% of the genes that encode proteins identified as possible TFP2 clients in *E. coli* have an orthologue in *Leptospirillum* sp. CF-1, suggesting that TFP2 is also an important player in proteostasis in this species. In addition, the H_2_O_2_ stress induced in *E. coli* represents an equivalent stress condition in *Leptospirillum* since concentrations in the range of 0.5–2 mM were able to induce an active response against oxidative stress [26]. Therefore, the functionality of TFP2 in members of the genus *Leptospirillum* could be linked to protection against H_2_O_2_ or other types of ROS.

## 3. Discussion

In this work, we postulated that the thioredoxin-fold protein TFP2 from *Leptospirillum* sp. CF-1 could have a role as chaperedoxin similar to the protein CnoX from *E. coli*. Under oxidant conditions generated by hypochlorous acid, the latter protein functions as a holdase that provides redox protection to its client proteins and promotes the refolding of unfolded substrates through interactions with the chaperone foldases DnaK and GroEL/ES [10]. Herein, the multiplex alignment showed that TFP2 conserves the CXXC motif (WCPGC) characteristic of thioredoxin-fold proteins. The cysteine residues of this motif are the key players in the thiol-disulfide exchange reaction [27]. In addition, the tryptophan residue that was highly conserved in *Leptospirillum* (and other acidophiles) may play a relevant role in the activity of TFP2. In thioredoxins from *E. coli* and *Salmonella* sp., this residue is not conserved (Figure 1B) [28]. However, in *Staphylococcus aureus* the replacement of this residue by alanine led to the formation of a domain-swapped dimer that was devoid of any of the biochemical activity known for Trx-fold proteins [29], thus indicating a pivotal role of this tryptophan residue in the Trx of this bacterium. The analysis of the sequence also showed the presence of conserved Pro 35–41, Phe 28, and Asp 27 residues, which are considered to possess key structural and functional roles [12,30,31]. On the other hand, the alignments revealed that TFP2 lacks the tetratricopeptide-repeat (TRP) domain characteristic of CnoX [10]. This domain increases the hydrophobicity and efficiency of the interaction with unfolded substrates. Therefore, its absence in TFP2 could hinder the interaction with client proteins [13]. Nevertheless, and according to our findings, the predicted product from the *hyp* gene adjacent to *tfp2* is a small hypothetical protein with low similarity to a segment of amino acids of the TRP domain of CnoX_Ec. Whether the Hyp protein or another protein with a TRP domain favors interactions of TFP2 with its unfolded substrates deserves to be experimentally addressed.

Like *Caulobacter* CnoX [24], our analysis showed that TFP2 exhibits thioredoxin activity, which could play a role in catalyzing the reduction of oxidized cysteine residues in cell proteins. Furthermore, it was observed that TFP2 could catalyze the reduction of oxidized methionine sulfoxide reductase MsrA, a well-known substrate of thioredoxin in a wide range of organisms [31], thus suggesting a role of TFP2 in the maintenance of cell proteostasis. Our results also indicate that oxidized TFP2 can be restored and recycled by the reductase activity of the NADPH-dependent flavoenzyme TrxR [32]. In addition, we determined that TFP2 possesses holdase chaperone activity that contributes towards avoiding aggregation of the thermolabile enzyme CS. Altogether, these results are consistent with the idea that TFP2 from the strain CF-1 is a chaperedoxin-like protein with holdase activity that provides redox protection to the cell.

According to our data, TFP2 increased the tolerance of *E. coli* to peroxide, but not to diamine or HOCl. In *E. coli*, HOCl turns CnoX into a holdase by chlorination of several residues in the TPR domain [10]. This mechanism permits the generation of an adequate and coordinated redox protection response against the harmful effect of HOCl, the major oxidant produced by neutrophils and other cells of the host immune system [33]. Since TFP2 from CF-1 lacks the C-terminal TRP domain, the insensitivity of this protein to HOCl is a predictable fact that is in line with our findings. In addition, *Leptospirillum* spp. is a free-living bacterium that is naturally exposed to external H_2_O_2_ generated on the surface of minerals [16,17,18] but is unlikely to encounter HOCl in its natural habitats. On the other hand, when cells are exposed to H_2_O_2_ or other ROS, sensitive cysteines of the cell proteins are first modified to sulfenic acids (-SOH) and further oxidized to disulfide. In our case, the damage caused to proteins upon exposure of *E. coli* to peroxide could have been reversed by their interaction with the overproduced TFP2, which, through its chaperedoxin-like activity, could not only reduce client proteins but also facilitate refolding and diminish aggregation of others. Whether TFP2 transfers its substrates to DnaK/J/GrpE and/or GroEL/ES and, in this way, cooperates with these refolding systems remains to be established.

The genetic context of TFP2 is particularly interesting. In the genome of *E. coli*, the *cnoX* gene is located 694,219 bp from *groEL*/*ES* and 410,021 bp from *dnaK*/*J*/*grpE*. On the other hand, in *Leptospirillum* sp. CF-1, GroEL/ES encoding genes are the direct neighbors of *tfp2*. Interestingly, all six genes of the “*tfp2* cluster” exhibit an increase in their mRNA levels in response to H_2_O_2_ treatment, suggesting a common regulatory pattern, and reinforcing the idea of collaboration between the chaperedoxin-like TFP2 and the foldase chaperone machinery GroEL/ES. In addition, the genes that encode the carboxy-terminal protease CtpA, and the recombination and DNA repair protein RecN, have roles in protection under environmental stress conditions [24,34,35,36]. In summary, the organization of the locus and the gene expression pattern under stress converts the “*tfp2* cluster” into an important player in the oxidative stress response in *Leptospirillum* spp.

In agreement with a chaperedoxin-like activity of TFP2, overexpression of *tfp2* in *E. coli* reduced the quantity of aggregated proteins, indicating its role as an antiaggregant factor. The proteomic analysis of the aggregates revealed a variety of proteins that totally or partially reduce their aggregation when TFP2 is overproduced, suggesting that they constitute possible client proteins of this chaperedoxin-like protein. The analysis allowed us to identify 124 proteins that belong to fifteen metabolic categories, including energy conversion, transport, and metabolism of different cellular components (amino acids, nucleotides, carbohydrates, coenzymes, and lipids), as well as some related to translation, transcription, proteostasis maintenance, and defense mechanisms. Although it is feasible that some of these proteins precipitate due to their abundance, such as the case of ribosomal proteins, the vast majority of them have a low tendency to aggregate, and it is possible that their aggregation is due to induced oxidative stress. In the same way, de-aggregation seems to be a direct effect of the presence of TFP2 inside the cell. A more detailed analysis of TFP2 client proteins in *Leptospirillum* will require more specific experimental approaches for analysis, such as co-immunoprecipitation experiments.

Finally, in agreement with cooperative functionality between chaperedoxin and holdase machinery, the bulk of predicted TFP2 client proteins are potential substrates (Table S1) of *E. coli* DnaK or GroEL/ES [37,38]. Furthermore, it is of note that most of the genes encoding these proteins have orthologues in the CF-1 strain. The role of TFP2 as an antiaggregation factor of specific proteins from the strain CF-1 should be explored in future research. In summary, in *Leptospirillum* spp. The antiaggregant chaperedoxin-like TFP2 could play a role of utmost importance in the redox protection of a number of proteins and, thus, in cellular proteostasis under the oxidative conditions that are habitual in its natural or industrial environments.

## 4. Materials and Methods

### 4.1. Structural Analysis

The alignment of the TFP2 protein from *Leptospirillum* sp. CF-1 with the sequence of CnoX from *E. coli* was performed (crystal structure 3QOU; [11] using Clustal Omega. Modelling of TFP2 was undertaken by Modeller using Chimera (https://www.rbvi.ucsf.edu/chimera) (accessed on 18 August 2023). Assessment and validation of the structural model were performed using Discrete Optimized Protein Energy score (DOPE) and default parameters of Swiss Model server (https://swissmodel.expasy.org/assess) (accessed on 6 May 2024).

### 4.2. Multiple Sequence Alignment Analysis

Amino-acid sequences were aligned using MUSCLE. The construction algorithm Maximum Likehood was added to a Bootstrap of 1000 integrated within the MEGA11 program version 11.0.9 [39]. The alignment was visualized using the software Jalview version 2.11.2.5 [40].

### 4.3. Bacterial Strains, Growth, and Viability

BW25113, TOP10, and BL21 *E. coli* strains were grown in LB medium at 37 °C with shaking. The strains (derived from the *E. coli* AG1 parental strain) that over-expressed cloned *trxA*, *msrA*, or *cnoX* genes obtained from the ASKA library [41] were grown in LB medium supplemented with 250 µg/mL chloramphenicol. The KEIO strain (derived from the *E. coli* K-12 parental strain), which is a mutant in the *cnoX* gene [42], was supplemented with 50 µg/mL kanamycin.

*Leptospirillum* sp. CF-1 was grown in 9K BR medium [0.132 g/L (NH_4_)_2_SO_4_; 0.0373 g/L KCl, 0.0087 g/L K_2_HPO_4_; 1.23 g/L MgSO_4_·H_2_O and 0.027 g/L Ca(NO)_3_·H_2_O; pH 1.75], containing ferrous sulfate [18.4 g/L FeSO_4_·7H_2_O (pH 1.2)] at 37 °C with constant agitation at 180 rpm [21].

### 4.4. Susceptibility Assay of E. coli to H_2_O_2_

*Minimal inhibitory concentration* (MIC). The WT, WT/pBAD, WT/pBAD-*tfp2*, *cnoX*(-)/pBAD and *cnoX*(-)/pBAD-*tfp2* strains were grown in TYES minimum medium at 37 °C until OD_600nm_ = 0.3. Plates of 96 wells were prepared with serial dilutions (1:2) of 0–200 mM H_2_O_2_, diamide, and HOCl in a final volume of 200 µL, after which L–arabinose to a final concentration of 0.2% *w*/*v*, 100 µg/mL ampicillin (if appropriate) and culture bacteria to a final concentration of 1% were added to each well. Growth was followed by measuring the absorbance for 24 h at 600 nm using a Tecan Infinite M200 multiplate reader.

*Growth curves*. For the genetic complementation experiments, the strains were grown in TYES minimum medium at 37 °C until OD_600nm_ = 0.3. L–arabinose was added to a final concentration of 0.2% *w*/*v*, and the cells were treated with 0.39 mM H_2_O_2_ (1/4 MIC). A control without oxidant was also included. Growth was followed by measuring the absorbance for 24 h, as indicated above.

*Cell viability*. For cell viability determination, after adding L–arabinose and H_2_O_2_ (0, 4, 8, or 16 mM) for 2 h, serial dilutions were prepared in 0.9% NaCl, and 4 µL drops were pipetted onto LB agar plates. Plates were incubated overnight at 37 °C.

### 4.5. Cloning in pet21b Expression Vector

The coding sequence of *tfp2* from *Leptospirillum* sp. CF-1 was amplified by PCR using wild-type genomic DNA as a template. The PCR reaction was carried out in a total volume of 50 µL using CF-1 DNA (1 µg), dNTPs (1 mM), forward and reverse primers (0.5 µM, Table 2), Q5 High-fidelity DNA Polymerase (0.02 U/µL, New England Biolabs M0491), Q5 Reaction Buffer (1X), and nuclease-free water (until 50 µL). The PCR reaction was performed using the manufacturer’s indications with Tm = 66 °C. The amplified *tfp2* gene and pet21b expression plasmid were purified and digested with *Nde*I and *Hind*III restriction enzymes following the instructions of the provider. The digested PCR product and plasmid were then ligated using T4 Ligase (Bioline) and standard procedures. Finally, 5 µL of the ligation mix was transformed into chemocompetent *E. coli* TOP10 and BL21 cells, which were then selected on LB-ampicillin plates as described in Section 4.3.

### 4.6. Protein Purification

Bacterial cultures were grown overnight and diluted 1:100 in fresh LB medium supplemented with chloramphenicol for the ASKA strains and ampicillin for the *E. coli* BL21_pet21b/tfp2 strain, as described in Section 2.3. Gene expression was induced overnight with the addition of 1 mM isopropyl-ß-D-1-thiogalactopyranoside (IPTG) to 1 L of cultures at OD_600nm_~0.3–0.4. Cultures were sedimented at 10,650× *g* for 5 min at 4 °C. The pellet was sonicated on ice in binding buffer (20 mM sodium phosphate pH 7.4, 0.5 M NaCl, and 20 mM imidazole) supplemented with 0.1 mM phenylmethylsulfonyl fluoride (PMSF, Roche). Cell debris was discarded at 10,595× *g* for 10 min at 4 °C and the supernatant was loaded onto a Ni^2+^ affinity column (HisTrap, GE Healthcare, Chicago, IL, USA). The column was washed with 20 column volumes (CV) of binding buffer, and the proteins were eluted with 5 CV of elution buffer (20 mM sodium phosphate pH 7.4, 0.5 M NaCl, and 0.5 M imidazole). The protein-containing fractions were concentrated in AMICON filters (cutoff: 10 kDa for MsrA_Ec and CnoX_Ec; 3 kDa for TrxA_Ec and TFP2_CF-1). The concentration of purified proteins was determined by the Bradford method [43] using bovine serum albumin (BSA) as standard.

### 4.7. Thioredoxin Activity Assay

Trx activity was measured using the insulin reduction assay according to [23]. Briefly, 150 µM insulin (Sigma-Aldrich, St. Louis, MO, USA) and 10 µM of tested protein (TrxA, CnoX, or TFP2) were mixed in 100 mM potassium phosphate (pH 7.0) and 1 mM EDTA. The reaction was initiated by adding a final concentration of 0.8 mM DTT. The reduction of insulin was monitored at 650 nm.

### 4.8. Thiol Reductase Assay

Thiol reductase activity was measured using the NADPH consumption assay. MsrA (200 µM) was oxidized with 40 mM diamide in 50 mM phosphate buffer (pH 7.4) for 30 min at 37 °C. The diamide was removed using a desalting column (Nap-5, GE Healthcare). Then 100 µM NADPH, 1.25 µM TrxR, and 5 µM TFP2 or CnoX were mixed until the slope corresponding to NADPH reduction had stabilized. Subsequently, 10 µM oxidized MrsA was added, and absorbance was followed at 340 nm. The activity was calculated as Δ340 nm/min.

### 4.9. Reduction of TFP2 by TrxR

TFP2 was oxidized with 40 mM diamide in 50 mM phosphate buffer (pH 7.4) for 30 min at 37 °C. Diamide was removed as mentioned earlier (Section 4.8): The reaction mixture contained 200 µM NADPH, 1.25 µM TrxR (#cat T9074 Sigma Aldrich, St. Louis, MO, USA), and oxidized TFP2 (0, 1, 3, and 5 µM). The activity was measured spectrophotometrically by monitoring the absorbance at 340 nm for 20 min.

### 4.10. Determination of Chaperone Activity

Chaperone activity was assayed in vitro using citrate synthase (CS), according to [44]. CS is a thermolabile protein that unfolds and aggregates when incubated at 43 °C (thermal aggregation). Briefly, 2 µM CS (Sigma-Aldrich, St. Louis, MO, USA) was incubated in 40 mM HEPES-KOH (pH 7.5) at 43 °C in the presence of 16 µM TFP2 or 6 µM in 1:1 ratio chaperone:CS. Aggregation was monitored by measuring light scattering at 360 nm over 15 h.

### 4.11. Cloning pBAD-tfp2 in E. coli

pBAD-*tfp2* and the empty vector [21] were introduced by electroporation into *E. coli* BW25113 and *cnoX*(-) strains. The transformants were selected in LB ampicillin, as described in Section 4.3.

### 4.12. Protein Aggregation Measurement

The quantification of protein aggregation was carried out according to the method published by [45] with slight modifications. Briefly, the cells were grown as mentioned above to OD_600nm_ = 0.3; then, the cultures were supplemented with L–arabinose (0.2% *w*/*v*) and incubated for 2 h with 4 mM H_2_O_2_. A control without oxidant was also included. Bacterial cultures (50 mL) were cooled on ice and centrifuged for 10 min at 5000× *g* at 4 °C. Pellets were resuspended in 250 µL buffer A (10 mM potassium phosphate buffer pH 6.5, 1 mM EDTA, 20% *w*/*v* sucrose and 1 mg/mL lysozyme) and incubated for 30 min on ice. Cell lysis was performed by the addition of 360 µL buffer B (10 mM potassium phosphate buffer pH 6.5, 1 mM EDTA) followed by sonication (8 cycles, 15 s, 60% amplitude) while cooling. Intact cells were removed by centrifugation at 4000 rpm for 15 min at 4 °C. The insoluble cell fraction (containing membranes and aggregated proteins) was isolated by subsequent centrifugation at 15,000× *g* for 20 min at 4 °C. The pellet fractions were frozen, resuspended in 400 µL buffer B by brief sonication, and centrifuged at 13,000 rpm for 20 min at 4 °C. The washed pellet fractions were again resuspended in 320 µL buffer B by brief sonication; afterwards, 80 µL NP40 (10% *v*/*v*) was added, and the aggregated proteins were isolated by centrifugation at 15,000× *g* for 30 min at 4 °C. This washing procedure was repeated three times to completely remove the contaminating membrane proteins. The pellets were resuspended in 200 µL buffer B by brief sonication, and finally, the aggregated proteins were resolved by 12% SDS-PAGE, visualized with Coomassie blue (Biorad^®^, Tokyo, Japan), and quantified as described above (Section 4.6).

### 4.13. Relative Expression of tfp2 and Neighboring Genes under Oxidative Conditions

Transcript levels of *tfp2* and neighboring genes were measured in CF-1 cells exposed to oxidative stress with 1 mM H_2_O_2_ as described previously [21]. Non-stressed cell cultures were used as controls. After stress induction, total RNA was extracted, and cDNA was synthesized from 1 µg of total RNA using a reverse transcription kit (#cat G592, ABM, Richmond, BC, Canada) according to the instructions of the provider. The cDNA was used to perform the qPCR experiments in a real-time thermal cycler (Applied Biosystems, Waltham, MA, USA). Three independent trials were averaged in all cases. The 16S rRNA encoding gene (*rrsB*) was used as a housekeeping gene to normalize gene expression.

### 4.14. Identification of TFP2 Client Proteins in E. coli

Cultures (50 mL) of the mutant *E. coli cnoX*(-) strain carrying the empty pBAD or pBAD-*tfp2* vector were exposed to 4 mM H_2_O_2_ for 2 h. Cells were then collected, and protein aggregates from each cell pellet were obtained, as indicated previously (Section 4.12). The aggregates were frozen in dry-ice ethanol and stored at −80 °C until use.

Mass spectrometry-based quantitative proteomic was performed using LC-MS/MS analysis with the EASY-nLC 1000 split-free UHPLC instrument coupled to an LTQ Orbitrap Velos Pro mass spectrometer (Thermo Fisher Scientific Inc., Waltham, MA, USA). Proteins were prepared as described previously [46]. Briefly, 50 µg of protein of each condition were tryptically cleaved using an S-trap procedure. Subsequently, peptides were purified and fractionated by sequential elution, and the samples of each replicate were analyzed. Briefly, peptides were separated using reversed phase chromatography with a binary gradient from 5 to 50% acetonitrile, and 0.1% acetic acid at a constant flow rate of 300 nL/min. Full survey scans were performed with a resolution of 60,000 in the range of 333–1650 *m*/*z*. MS/MS scans were taken for the fifteen most abundant precursor ions per scan cycle, excluding unassigned charge states and singly charged ions, and dynamic exclusion was enabled for 30 s. Internal lock mass calibration was applied (lock mass 445.12003). A database search and label-free quantification (LFQ) was carried out using the MaxQuant proteomics software package version 1.6.10.43 [47]. A protein sequence database for *E. coli* BW25113 with 4298 entries downloaded from NCBI (20 February 2023), and common contaminants and reverse sequences added by the MaxQuant software were used for protein identification. The parameters were set as described by [46]. *E. coli* proteins detected in at least two replicates of at least one of the conditions were considered identified. The MaxQuant companion software Perseus version 1.6.15.0 [47] was used for data analysis. Averaged LFQ intensities were used to calculate log2 fold changes.

The data analysis, which comprehends data transformation, normalization, and statistics, was performed using the Real Statistics v8.8 [48] resource pack on Microsoft Excel 35 v2307, as described in [49]. The plots were generated by R v4.3.0 on RStudio v2023.03.1 [50,51] using inhouse code and the libraries ggrepel, ggplot2, tidyverse, hrbthemes, tm, proustr, and VennDiagram. The analyses of predicted TFP2 client proteins were made using bioinformatic resources available in the Protein Homeostatis Database (http://phdb.switchlab.org/#/home?limit=10&page=1) (accessed on 18 May 2023). The aggregation propensity was estimated using Waltz and Tango computational programs that predict amyloid-forming regions in amino acid sequences [52].

### 4.15. Statistical Analysis

Experiments were carried out in triplicate. One-way ANOVA with multiple comparisons (enzymatic and aggregation assays) and Student’s *t*-test (qRT-PCR) statistical analyses were performed using Graphpad Prism version 10.0.1.

## 5. Conclusions

TFP2 from *Leptospirillum* sp. CF-1 is a chaperedoxin-like protein that conserves thioredoxin and holdase activities and prevents protein aggregation in response to hydrogen peroxide exposure without prior HOCl activation. The co-localization of the *tfp2* gene with genes encoding the GroEL/ES foldase chaperone system suggests a functional relationship of cooperation, although this still awaits experimental elucidation. The proteomic study showed that TFP2 acts on over a hundred client proteins, which perform a wide variety of cellular functions, reinforcing its relevance in the maintenance of cellular proteostasis in this extreme acidophilic bioleaching bacterium.

## Figures and Tables

**Table 1:** Aggregated proteins overrepresented in 1 mM H_2_O_2_-treated cells carrying empty vector. Listed proteins are grouped by COG category.

Uniprot ID	Protein	Gene	Log2 Fold Change	*p*-Value
C. Energy production and conversion				
P36683	bifunctional aconitate hydratase AcnB	*acnB*	6.77	0.015
P77252	putative lactate utilization oxidoreductase YkgE	*ykgE*	4.59	0.021
P0AFG3	subunit of E1(0) component of 2-oxoglutarate dehydrogenase	*sucA*	1.08	0.034
P0AFG6	dihydrolipoyltranssuccinylase	*sucB*	6.63	0.045
P09373	pyruvate formate-lyase	*pflB*	7.94	0.000
P0AC33	fumarase A	*fumA*	9.99	0.002
P0A9C0	anaerobic glycerol-3-phosphate dehydrogenase subunit A	*glpA*	2.34	0.037
P33602	NADH:quinone oxidoreductase subunit G	*nuoG*	4.45	0.049
P0AFC7	NADH:quinone oxidoreductase subunit B	*nuoB*	3.70	0.013
P61889	malate dehydrogenase	*mdh*	8.27	0.003
P22259	phosphoenolpyruvate carboxykinase (ATP)	*pckA*	6.48	0.001
P0ABB4	ATP synthase F1 complex subunit beta	*atpD*	7.32	0.004
P0ABA6	ATP synthase F1 complex subunit gamma	*atpG*	5.33	0.003
P0ABA4	ATP synthase F1 complex subunit delta	*atpH*	4.71	0.027
P0A9G6	isocitrate lyase	*aceA*	7.77	0.038
P00363	fumarate reductase flavoprotein subunit	*frdA*	5.29	0.045
E. Amino acid transport and metabolism				
P00968	carbamoyl-phosphate synthetase large subunit	*carB*	2.63	0.003
P30750	L-methionine/D-methionine ABC transporter ATP binding subunit	*metN*	4.13	0.049
P15288	peptidase D	*pepD*	3.60	0.027
P00509	aspartate aminotransferase	*aspC*	5.12	0.012
P04825	aminopeptidase N	*pepN*	6.49	0.006
P75914	zinc-binding phosphatase YcdX	*ycdX*	4.92	0.040
P16095	L-serine deaminase I	*sdaA*	7.14	0.003
P0A9S3	galactitol-1-phosphate 5-dehydrogenase	*gatD*	5.87	0.026
P07913	threonine dehydrogenase	*tdh*	7.38	0.004
P08142	acetohydroxy acid synthase I subunit IlvB	*ilvB*	5.96	0.004
P00963	asparagine synthetase A	*asnA*	7.40	0.026
P0AB80	branched-chain-amino-acid aminotransferase	*ilvE*	3.91	0.043
F. Nucleotide transport and metabolism				
P22333	adenosine deaminase	*add*	5.63	0.019
P0A8F0	uracil phosphoribosyltransferase	*upp*	7.77	0.024
P0A763	nucleoside diphosphate kinase	*ndk*	5.03	0.004
P0A7E5	CTP synthetase	*pyrG*	1.65	0.017
G. Carbohydrate transport and metabolism				
P69797	mannose-specific PTS enzyme IIAB component	*manX*	7.37	0.000
P37188	galactitol-specific PTS enzyme IIB component	*gatB*	9.14	0.002
P0C8J8	putative tagatose-1.6-bisphosphate aldolase 2 chaperone	*gatZ*	9.20	0.003
P0C8J6	tagatose-1.6-bisphosphate aldolase 2	*gatY*	9.59	0.001
P39829	galactarate dehydratase GarD	*garD*	4.30	0.032
P04983	ribose ABC transporter ATP binding subunit	*rbsA*	6.05	0.018
P0A9C9	fructose-1.6-bisphosphatase 2	*glpX*	4.71	0.010
P36672	trehalose-specific PTS enzyme IIBC component	*treB*	7.67	0.004
P0A6K6	phosphopentomutase	*deoB*	5.02	0.027
H. Coenzyme transport and metabolism				
P76085	phenylacetate-CoA ligase	*paaK*	5.49	0.049
P0A8Y1	pyrimidine 5-nucleotidase YjjG	*yjjG*	3.95	0.017
I. Lipid transport and metabolism				
P0AEK2	3-oxoacyl-[acyl-carrier-protein] reductase FabG	*fabG*	7.02	0.015
P0AAI5	3-oxoacyl-[acyl carrier protein] synthase 2	*fabF*	5.43	0.011
P0AEK4	enoyl-[acyl-carrier-protein] reductase	*fabI*	7.56	0.048
P0A9Q5	acetyl-CoA carboxyltransferase subunit beta	*accD*	4.80	0.038
P37440	oxidoreductase UcpA	*ucpA*	1.55	0.039
P. Inorganic ion transport and metabolism				
P13036	ferric citrate outer membrane transporter	*fecA*	4.28	0.009
J. Translation. ribosomal structure and biogenesis				
P0A8I8	23S rRNA m(3)psi1915 methyltransferase	*rlmH*	4.88	0.014
P0AEI1	isopentenyl-adenosine A37 tRNA methylthiolase	*miaB*	7.01	0.020
P0AEI4	ribosomal protein S12 methylthiotransferase RimO	*rimO*	7.88	0.006
P75838	ribosomal protein S12 methylthiotransferase accessory factor YcaO	*ycaO*	3.38	0.047
P0AG67	30S ribosomal subunit protein S1	*rpsA*	5.55	0.027
P0ABU2	redox-responsive ATPase YchF	*ychF*	7.45	0.007
P0A7I0	peptide chain release factor RF1	*prfA*	4.40	0.006
P0A7L3	50S ribosomal subunit protein L20	*rplT*	6.56	0.008
P39199	ribosomal protein L3 N(5)-glutamine methyltransferase	*prmB*	2.82	0.024
P0A6P5	50S ribosomal subunit stability factor	*der*	2.50	0.027
P0A7K6	50S ribosomal subunit protein L19	*rplS*	4.46	0.049
P00957	alanine--tRNA ligase/DNA-binding transcriptional repressor	*alaS*	3.31	0.031
P07012	peptide chain release factor RF2	*prfB*	7.24	0.005
P05055	polynucleotide phosphorylase	*pnp*	2.95	0.036
P0A705	translation initiation factor IF-2beta	*infB*	8.08	0.003
P0C0R7	23S rRNA 2-O-ribose U2552 methyltransferase	*rlmE*	4.16	0.037
P0A7X3	30S ribosomal subunit protein S9	*rpsI*	8.27	0.036
P0A6K3	peptide deformylase	*def*	4.92	0.027
P0AG44	50S ribosomal subunit protein L17	*rplQ*	5.86	0.044
P0A7S9	30S ribosomal subunit protein S13	*rpsM*	6.68	0.028
P0AG55	50S ribosomal subunit protein L6	*rplF*	3.05	0.049
P0A7W7	30S ribosomal subunit protein S8	*rpsH*	4.59	0.016
P62399	50S ribosomal subunit protein L5	*rplE*	3.92	0.010
P0A7V3	30S ribosomal subunit protein S3	*rpsC*	9.26	0.021
P61175	50S ribosomal subunit protein L22	*rplV*	7.24	0.024
P0A7U3	30S ribosomal subunit protein S19	*rpsS*	7.63	0.016
P60422	50S ribosomal subunit protein L2	*rplB*	8.76	0.006
P0ADZ0	50S ribosomal subunit protein L23	*rplW*	6.85	0.021
P60438	50S ribosomal subunit protein L3	*rplC*	6.31	0.002
P0A6M8	elongation factor G	*fusA*	6.81	0.024
P0A6U3	5-carboxymethylaminomethyluridine-tRNA synthase subunit MnmG	*mnmG*	5.09	0.027
P0CE48	translation elongation factor Tu 2	*tufB*	7.05	0.017
P0A7J7	50S ribosomal subunit protein L11	*rplK*	9.30	0.024
P0A7L0	50S ribosomal subunit protein L1	*rplA*	3.64	0.006
K. Transcription				
P60240	RNA polymerase-binding ATPase and RNAP recycling factor	*rapA*	6.78	0.000
P0A972	transcription antiterminator and regulator of RNA stability CspE	*cspE*	4.78	0.027
P0A9F3	DNA-binding transcriptional dual regulator CysB	*cysB*	3.15	0.039
P07604	DNA-binding transcriptional dual regulator TyrR	*tyrR*	4.31	0.016
P0ACM2	DNA-binding transcriptional repressor RspR	*rspR*	3.77	0.014
P31802	DNA-binding transcriptional dual regulator NarP	*narP*	4.35	0.015
P0AA16	DNA-binding transcriptional dual regulator OmpR	*ompR*	1.62	0.007
P46837	putative RNA-binding protein YhgF	*yhgF*	6.70	0.000
P06993	DNA-binding transcriptional activator MalT	*malT*	6.59	0.006
P0AFG0	transcription termination/antitermination factor NusG	*nusG*	5.00	0.039
P0A8V2	RNA polymerase subunit beta	*rpoB*	6.38	0.043
L. Replication. recombination and repair				
P0A812	Holliday junction branch migration complex subunit RuvB	*ruvB*	5.03	0.012
D. Cell cycle control. cell division. chromosome partitioning				
P0AEZ3	Z-ring positioning protein MinD	*minD*	8.03	0.009
M. Cell wall/membrane/envelope biogenesis				
P17952	UDP-N-acetylmuramate--L-alanine ligase	*murC*	1.90	0.021
P02931	outer membrane porin F	*ompF*	6.44	0.020
P0A910	outer membrane protein A	*ompA*	4.04	0.020
P0AEP3	UTP--glucose-1-phosphate uridylyltransferase	*galU*	8.19	0.000
P69776	murein lipoprotein	*lpp*	6.21	0.040
P37751	putative glycosyltransferase WbbK	*wbbK*	5.64	0.000
P37749	beta-1.6-galactofuranosyltransferase WbbI	*wbbI*	5.10	0.023
P37747	UDP-galactopyranose mutase	*glf*	6.24	0.003
P06996	outer membrane porin C	*ompC*	7.27	0.047
P02930	outer membrane channel TolC	*tolC*	2.90	0.023
P0A749	UDP-N-acetylglucosamine 1-carboxyvinyltransferase	*murA*	2.32	0.002
P0A9V1	lipopolysaccharide transport system ATP binding protein LptB	*lptB*	4.70	0.023
P25714	membrane protein insertase YidC	*yidC*	4.86	0.014
P17169	L-glutamine-D-fructose-6-phosphate aminotransferase	*glmS*	4.88	0.018
P22634	glutamate racemase	*murI*	4.40	0.025
O. Posttranslational modification. protein turnover. chaperones				
P0ABZ6	chaperone SurA	*surA*	5.40	0.032
P0ACA7	glutathione S-transferase GstB	*gstB*	3.75	0.012
P63284	chaperone protein ClpB	*clpB*	3.70	0.042
P0AAI3	ATP-dependent zinc metalloprotease FtsH	*ftsH*	2.44	0.017
P39099	periplasmic serine endoprotease	*degQ*	3.77	0.002
P0A6F5	chaperonin GroEL	*groEL*	2.27	0.018
P0ABC3	regulator of FtsH protease	*hflC*	2.21	0.038
T. Signal transduction mechanisms				
P0A964	chemotaxis protein CheW	*cheW*	5.91	0.011
V. Defense mechanisms				
P0AE08	alkyl hydroperoxide reductase. AhpC component	*ahpC*	6.43	0.033
P0ABT2	DNA protection during starvation protein	*dps*	1.86	0.010
P06610	thioredoxin/glutathione peroxidase BtuE	*btuE*	4.96	0.026
Poorly Characterized				
R. General function prediction only				
P24203	P-loop guanosine triphosphatase YjiA	*yjiA*	5.61	0.007
Non categories				
P0DTT0	50S ribosomal subunit assembly factor BipA	*bipA*	2.76	0.038

**Table 2:** Primers used in this work.

Gene	GenBank Code	Primer Name	Sequence (5′-3′)	Amplicon (bp)
		Pet21b cloning		
*tfp2*	AKS23718	pET21b_F_TFP2	TTCCATATGGTGGAAGTAAATGCTCCG	315
		pET21b_R_TFP2	TTTAAGCTTGGACTTGAGAAGAGAGTCAA	
		RT-qPCR		
*proP*	AKS24793	Prot_RT2_F	AGACCCTATCTTCACTTCCC	105
		Prot_RT2_R	AATCGTCGCTCTCTTGTAATC	
*groES*	AKS23720	GroES_RT2_F	CAGAAAGGCAAGATCGAAAG	96
		GroES_RT2_R	TGTGATCTTGGAACCAGAATA	
*groEL*	AKS23719	GroEL_RT2_F	CGTGGCTATATCTCTCCCTAT	101
		GroEL_RT2_R	ATGCTGCTGACTTTCTTCTC	
*hyp*	*	Hyp_RT2_F	TTAATTCTCTTCTGGCCTACA	108
		Hyp_RT2-R	TTCTTGAATTCTTTGTATCGGA	
*tfp2*	AKS23718	pTfp2-F	CTGGTAATGGTTGATTTCTGGG	104
		pTfp2-R	GGAATGCCCATGACCTGATAT	
*recN*	AKS23717	RecN_RT_F	AGCAGCAAGTATGGTGTATG	97
		RecN_RT_R	TACTTTCAGACGGTCCAAATC	
*rrsB*	CP012147	pRrsB-F	TACAAGCTTCCGCTCCTG	288
		pRrsB-R	CCGGGCAAAAGTGGTTTACA	

F: forward; R: reverse. (*): non-annotated gene (location: 1.593.519–1.593.662).

## Data Availability

Data is contained within the article and Supplementary Material.

## Supplementary Materials

The following supporting information can be downloaded at: https://www.mdpi.com/article/10.3390/ijms25136905/s1.
