# Correction: Muñoz-Villagrán et al. The Thioredoxin Fold Protein (TFP2) from Extreme Acidophilic *Leptospirillum* sp. CF-1 Is a Chaperedoxin-like Protein That Prevents the Aggregation of Proteins under Oxidative Stress. *Int. J. Mol. Sci.* 2024, *25*, 6905

**DOI:** 10.3390/ijms252312489

**Published:** 2024-11-21

**Authors:** Claudia Muñoz-Villagrán, Javiera Acevedo-Arbunic, Elisabeth Härtig, Susanne Sievers, Daniela Zühlke, Francisco Issotta, Carolina Mascayano, Dieter Jahn, Martina Jahn, Gloria Levicán

**Affiliations:** 1 Laboratorio de Microbiología Básica y Aplicada, Departamento de Biología, Facultad de Química y Biología, Universidad de Santiago de Chile (USACH), Santiago 9170022, Chile; 2 Institute of Microbiology, Technische Universität Braunschweig, Spielmannstr 7, 38106 Braunschweig, Germany; e.haertig@tu-braunschweig.de (E.H.);; 3 Department of Microbial Physiology and Molecular Biology, Institute of Microbiology, University of Greifswald, 17489 Greifswald, Germanydaniela.zuehlke@uni-greifswald.de (D.Z.); 4 Departamento Genética Molecular y Microbiología, Facultad de Ciencias Biológicas, Pontificia Universidad Católica, Santiago 8331150, Chile; 5 Laboratorio de Simulación Computacional y Diseño Racional de Fármacos, Departamento de Ciencias del Ambiente, Facultad de Química y Biología, Universidad de Santiago de Chile (USACH), Santiago 9170022, Chile; 6 Braunschweig Integrated Centre of Systems Biology BRICS, Technische Universität Braunschweig, Rebenring 56, 38106 Braunschweig, Germany

Susanne Sievers and Daniela Zühlke were not included as authors in the original publication [1]. These authors contributed to the published research article by performing the proteomics experiments shown in the manuscript (Section 2.5, Table 1). These authors were missing from the published version due to a communication error made by the authors during the final stages of manuscript preparation. Due to the addition of these missing authors, a new institution, “Department of Microbial Physiology and Molecular Biology, Institute of Microbiology, University of Greifswald, 17489 Greifswald, Germany”, has also been added to the affiliation list. The corrected Author Contributions statement appears below. The authors state that the scientific conclusions are unaffected. This correction was approved by the Academic Editor. The original publication has also been updated.
