# Supplementary material for: The Thioredoxin Fold Protein (TFP2) from Extreme Acidophilic Leptospirillum sp. CF-1 Is a Chaperedoxin-like Protein That Prevents the Aggregation of Proteins under Oxidative Stress
Source: Int J Mol Sci. 2024 Jun 24;25(13):6905. doi: 10.3390/ijms25136905 (PMC11241051; doi:10.3390/ijms25136905)
Supplement: Supplementary file 1 [file ijms-25-06905-s001.zip › Table S1.pdf]

Table S1. Characteristics of aggregated proteins overrepresented in 1 mM H_2O_2-treated cells carrying empty vector. Listed proteins are grouped by COG category

				Intrinsic Aggregation Propensity		
Uniprot	Protein	Gene	%Cys	TANGO score (*)	WALTZ score (*)	Chaperone client classification (**)
**C, Energy production and conversion**						
P36683	bifunctional aconitate hydratase AcnB	*acnB*	1.16	3.19	2.09	TKG
P77252	putative lactate utilization oxidoreductase YkgE	*ykgE*	3.77	5.11	0.84	KG
P0AFG3	subunit of E1(0) component of 2-oxoglutarate dehydrogenase	*sucA*	1.07	1.45	2.81	TKG
P0AFG6	dihydrolipoyltranssuccinylase	*sucB*	0	2.9	1.8	TKG
P09373	pyruvate formate-lyase	*pflB*	0.66	2.81	2.23	TKG
P0AC33	fumarase A	*fumA*	1.64	2.75	1	TKG
P0A9C0	anaerobic glycerol-3-phosphate dehydrogenase subunit A	*glpA*	2.58	1.89	1.63	KG
P33602	NADH:quinone oxidoreductase subunit G	*nuoG*	1.76	2.07	1.18	TKG
P0AFC7	NADH:quinone oxidoreductase subunit B	*nuoB*	2.27	3.18	1.42	TKG
P61889	malate dehydrogenase	*mdh*	0.96	3.47	1.44	TKG
P22259	phosphoenolpyruvate carboxykinase (ATP)	*pckA*	0.93	3.22	3.23	TKG
P0ABB4	ATP synthase F1 complex subunit beta	*atpD*	0.22	5.45	0.56	KG
P0ABA6	ATP synthase F1 complex subunit gamma	*atpG*	0.7	1.9	3.47	K
P0ABA4	ATP synthase F1 complex subunit delta	*atpH*	1.13	3.42	1.53	K
P0A9G6	isocitrate lyase	*aceA*	1.15	1.59	1.66	S
P00363	fumarate reductase flavoprotein subunit	*frdA*	1.66	1.07	2.3	TKG
**E, Amino acid transport and metabolism**						
P00968	carbamoyl-phosphate synthetase large subunit	*carB*	1.3	1.44	1.18	TKG
P30750	L-methionine/D-methionine ABC transporter ATP binding subunit	*metN*	1.46	3.2	1.37	KG
P15288	peptidase D	*pepD*	0.82	3.66	2.7	TK
P00509	aspartate aminotransferase	*aspC*	1.26	3.59	4.26	TKG
P04825	aminopeptidase N	*pepN*	0.92	3.95	3.61	TKG
P75914	zinc-binding phosphatase YcdX	*ycdX*	1.22	2.13	3.08	TKG
P16095	L-serine deaminase I	*sdaA*	1.98	2.08	1.75	KG
P0A9S3	galactitol-1-phosphate 5-dehydrogenase	*gatD*	2.31	4.16	2.33	TKG
P07913	threonine dehydrogenase	*tdh*	1.76	1.26	1	TKG
P08142	acetohydroxy acid synthase I subunit IlvB	*ilvB*	1.6	1.96	3.71	TKG
P00963	asparagine synthetase A	*asnA*	0.61	2.59	0.14	TKG
P0AB80	branched-chain-amino-acid aminotransferase	*ilvE*	0.97	2.98	4.19	TKG
**F, Nucleotide transport and metabolism**						
P22333	adenosine deaminase	*add*	1.2	1.22	1.59	KG
P0A8F0	uracil phosphoribosyltransferase	*upp*	0.48	4.09	0.49	KG
P0A763	nucleoside diphosphate kinase	*ndk*	0.7	6.7	5.42	TKG
P0A7E5	CTP synthetase	*pyrG*	1.47	3.9	1.87	TKG
**G, Carbohydrate transport and metabolism**						

P69797	mannose-specific PTS enzyme IIAB component	*manX*	0	7.28	0.63	TKG
P37188	galactitol-specific PTS enzyme IIB component	*gatB*	4.26	5.28	0.51	TKG
P0C8J8	putative tagatose-1,6-bisphosphate aldolase 2 chaperone	*gatZ*	1.9	5.08	1.62	TKG
P0C8J6	tagatose-1,6-bisphosphate aldolase 2	*gatY*	1.41	1.98	3.04	TKG
P39829	galactarate dehydratase GarD	*garD*	1.34	5.28	1.4	N
P04983	ribose ABC transporter ATP binding subunit	*rbsA*	0.4	3.65	4.93	TK
P0A9C9	fructose-1,6-bisphosphatase 2	*glpX*	0.89	5.57	4.64	TKG
P36672	trehalose-specific PTS enzyme IIBC component	*treB*	1.27	16.54	4.8	TK
P0A6K6	phosphopentomutase	*deoB*	1.47	3.38	0.76	TKG
**H, Coenzyme transport and metabolism**						
P76085	phenylacetate-CoA ligase	*paaK*	1.83	1.67	0.39	G
P0A8Y1	pyrimidine 5-nucleotidase YjjG	*yjjG*	1.33	0.98	4.25	TK
**I, Lipid transport and metabolism**						
P0AEK2	3-oxoacyl-[acyl-carrier-protein] reductase FabG	*fabG*	0	3.25	3.5	TKG
P0AAI5	3-oxoacyl-[acyl carrier protein] synthase 2	*fabF*	0.97	2.75	1.36	TKG
P0AEK4	enoyl-[acyl-carrier-protein] reductase	*fabI*	1.53	2.03	1.65	TKG
P0A9Q5	acetyl-CoA carboxyltransferase subunit beta	*accD*	1.97	1.67	4.62	K
P37440	oxidoreductase UcpA	*ucpA*	1.52	1	2.61	TKG
**P, Inorganic ion transport and metabolism**						
P13036	ferric citrate outer membrane transporter	*fecA*	0	1.72	5.07	N
**J, Translation, ribosomal structure and biogenesis**						
P0A8I8	23S rRNA m(3)psi1915 methyltransferase	*rlmH*	0.65	2.09	0.13	TKG
P0AEI1	isopentenyl-adenosine A37 tRNA methylthiolase	*miaB*	1.27	1.83	3.82	TKG
P0AEI4	ribosomal protein S12 methylthiotransferase RimO	*rimO*	2.04	2.1	1.01	TK
P75838	ribosomal protein S12 methylthiotransferase accessory factor YcaO	*ycaO*	1.19	3.2	2.79	TK
P0AG67	30S ribosomal subunit protein S1	*rpsA*	0.36	2.75	0.39	TKG
P0ABU2	redox-responsive ATPase YchF	*ychF*	1.65	0.8	2.83	KG
P0A7I0	peptide chain release factor RF1	*prfA*	0.83	0.21	0.14	TKG
P0A7L3	50S ribosomal subunit protein L20	*rplT*	0	7.72	4.29	TK
P39199	ribosomal protein L3 N(5)-glutamine methyltransferase	*prmB*	1.61	1.05	2.28	TKG
P0A6P5	50S ribosomal subunit stability factor	*der*	0.2	5.96	1.5	TK
P0A7K6	50S ribosomal subunit protein L19	*rplS*	0	6.23	0.29	TKG
P00957	alanine--tRNA ligase/DNA-binding transcriptional repressor	*alaS*	0.68	4.34	1.02	TKG
P07012	peptide chain release factor RF2	*prfB*	0.55	0.31	0.37	TK
P05055	polynucleotide phosphorylase	*pnp*	0.14	4.32	3.44	TKG
P0A705	translation initiation factor IF-2beta	*infB*	0.34	3.55	2.63	TKG
P0C0R7	23S rRNA 2-O-ribose U2552 methyltransferase	*rlmE*	0.96	3.74	0.77	TKG

P0A7X3	30S ribosomal subunit protein S9	*rpsI*	0	2.72	1.85	TKG
P0A6K3	peptide deformylase	*def*	1.18	1.41	3.85	TK
P0AG44	50S ribosomal subunit protein L17	*rplQ*	0.79	0.88	0.04	TK
P0A7S9	30S ribosomal subunit protein S13	*rpsM*	0.85	6.72	0.09	TKG
P0AG55	50S ribosomal subunit protein L6	*rplF*	0.56	1.2	1.61	TKG
P0A7W7	30S ribosomal subunit protein S8	*rpsH*	0.77	5.27	0.12	TKG
P62399	50S ribosomal subunit protein L5	*rplE*	0.56	2.66	3.89	TKG
P0A7V3	30S ribosomal subunit protein S3	*rpsC*	0	0.28	4.04	TKG
P61175	50S ribosomal subunit protein L22	*rplV*	0	4.73	0.39	TKG
P0A7U3	30S ribosomal subunit protein S19	*rpsS*	0	3.64	0.01	TK
P60422	50S ribosomal subunit protein L2	*rplB*	0.73	3.28	0.92	TKG
P0ADZ0	50S ribosomal subunit protein L23	*rplW*	0	5.97	0.01	TKG
P60438	50S ribosomal subunit protein L3	*rplC*	0	0.15	0.22	TKG
P0A6M8	elongation factor G	*fusA*	0.43	3.02	1.7	TKG
P0A6U3	5-carboxymethylaminomethyluridine-tRNA synthase subunit MnmG	*mnmG*	0.64	4.19	1.36	TKG
P0CE48	translation elongation factor Tu 2	*tufB*	0.76	5.88	1.34	TKG
P0A7J7	50S ribosomal subunit protein L11	*rplK*	0.7	5.32	3.36	TKG
P0A7L0	50S ribosomal subunit protein L1	*rplA*	0	1.88	0.84	TKG
**K, Transcription**						
P60240	RNA polymerase-binding ATPase and RNAP recycling factor	*rapA*	0.62	3.32	2.33	TKG
P0A972	transcription antiterminator and regulator of RNA stability CspE	*cspE*	0	4.97	7.97	TK
P0A9F3	DNA-binding transcriptional dual regulator CysB	*cysB*	0.31	4.32	4.03	TKG
P07604	DNA-binding transcriptional dual regulator TyrR	*tyrR*	1.17	2	2.46	TK
P0ACM2	DNA-binding transcriptional repressor RspR	*rspR*	1.75	1.55	1.45	TKG
P31802	DNA-binding transcriptional dual regulator NarP	*narP*	0	8.13	3.62	TK
P0AA16	DNA-binding transcriptional dual regulator OmpR	*ompR*	0.42	6.09	0.14	KG
P46837	putative RNA-binding protein YhgF	*yhgF*	0.65	0.79	1.82	N
P06993	DNA-binding transcriptional activator MalT	*malT*	1	5.91	3.62	TKG
P0AFG0	transcription termination/antitermination factor NusG	*nusG*	0	5.26	0.17	KG
P0A8V2	RNA polymerase subunit beta	*rpoB*	0.52	0.98	2.33	TKG
**L, Replication, recombination and repair**						
P0A812	Holliday junction branch migration complex subunit RuvB	*ruvB*	0	2.12	3.81	TKG
**D, Cell cycle control, cell division, chromosome partitioning**						
P0AEZ3	Z-ring positioning protein MinD	*minD*	0.74	4.77	2.24	TKG
**M, Cell wall/membrane/envelope biogenesis**						
P17952	UDP-N-acetylmuramate--L-alanine ligase	*murC*	0.41	3.87	1.64	TKG
P02931	outer membrane porin F	*ompF*	0	4.77	8.44	TKG
P0A910	outer membrane protein A	*ompA*	0.58	5.72	4.11	TKG
P0AEP3	UTP--glucose-1-phosphate uridylyltransferase	*galU*	1.66	3.18	1.76	TKG

P69776	murein lipoprotein	*lpp*	1.28	11.21	0.01	TKG
P37751	putative glycosyltransferase WbbK	*wbbK*	1.34	9.74	4.06	TKG
P37749	beta-1,6-galactofuranosyltransferase WbbI	*wbbI*	1.52	3.07	3.71	TKG
P37747	UDP-galactopyranose mutase	*glf*	0.54	1.53	5.6	TKG
P06996	outer membrane porin C	*ompC*	0	3.43	4.89	TKG
P02930	outer membrane channel TolC	*tolC*	0	2.72	5.69	TKG
P0A749	UDP-N-acetylglucosamine 1-carboxyvinyltransferase	*murA*	1.43	5.70	2.06	TKG
P0A9V1	lipopolysaccharide transport system ATP binding protein LptB	*lptB*	0.41	4.26	1.2	KG
P25714	membrane protein insertase YidC	*yidC*	0.18	14.07	3.92	K
P17169	L-glutamine--D-fructose-6-phosphate aminotransferase	*glmS*	0.66	3.59	0.73	TK
P22634	glutamate racemase	*murI*	1.75	8.1	1.33	TKG
**O, Posttranslational modification, protein turnover, chaperones**						
P0ABZ6	chaperone SurA	*surA*	0	2.48	2.97	TK
P0ACA7	glutathione S-transferase GstB	*gstB*	0.96	6.69	2.68	TKG
P63284	chaperone protein ClpB	*clpB*	0.35	3.88	1.24	TKG
P0AAI3	ATP-dependent zinc metalloprotease FtsH	*ftsH*	0.31	6.69	0.93	TKG
P39099	periplasmic serine endoprotease	*degQ*	0	4.27	3.57	G
P0A6F5	chaperonin GroEL	*groEL*	0.55	3.37	0.86	TKG
P0ABC3	regulator of FtsH protease	*hflC*	0	6.74	0.95	K
**T, Signal transduction mechanisms**						
P0A964	chemotaxis protein CheW	*cheW*	0	13.63	0.52	N
**V, Defense mechanisms**						
P0AE08	alkyl hydroperoxide reductase, AhpC component	*ahpC*	1.07	3.96	2.87	TKG
P0ABT2	DNA protection during starvation protein	*dps*	0	5.72	4.14	TKG
P06610	thioredoxin/glutathione peroxidase BtuE	*btuE*	1.64	7.13	3.01	TKG
**POORLY CHARACTERIED**						
**R, General function prediction only**						
P24203	P-loop guanosine triphosphatase YjiA	*yjiA*	1.57	6.04	3.38	TKG
**Non categories**						
P0DTT0	50S ribosomal subunit assembly factor BipA	*bipA*	0.66	4.36	3.98	TKG

Proteins highlighted in purple color represent unique proteins aggregated in ev_Ec-cells, but not in *tfp2*_Ec-cells.

*normalized by protein length.

** Chaperone client classification: **G**, GroEl/ES; **N**, proteins showing no chaperone dependence in any of the experiments; **K**, DnaK/J; **S**, contradictory results (i.e. increased mRNA or protein abundance upon chaperone deletion, and no other apparent chaperone dependencies in any of the studies); **T**, Trigger Factor. (Ramakrishnan et al., 2019).

Bioinformatic resources are available in Protein Homeostatis Database http://phdb.switchlab.org/#/home?limit=10&page=1
