# Supplementary material for: The Thioredoxin Fold Protein (TFP2) from Extreme Acidophilic Leptospirillum sp. CF-1 Is a Chaperedoxin-like Protein That Prevents the Aggregation of Proteins under Oxidative Stress
Source: Int J Mol Sci. 2024 Jun 24;25(13):6905. doi: 10.3390/ijms25136905 (PMC11241051; doi:10.3390/ijms25136905)
Supplement: Supplementary file 1 [file ijms-25-06905-s001.zip › Table S2.pdf]

Table S2. Characteristics of aggregated proteins overrepresented in 1 mM H2O2-treated cells carrying *tfp2* vector. Listed proteins are group by COG category.

				Intrinsic Aggregation Propensity		
**Uniprot**	**Protein**	**Gene**	**% Cys**	**TANGO score (*)**	**WALTZ score (*)**	**Chaperone client classification (**)**
**C, Energy production and conversion**						
P11349	Nitrate reductase A. subunit beta	*narH*	3.32	1.5	4.1	TK
P41407	FMN dependent NADH:quinone oxidoreductase	*azoR*	0	9.4	2.4	TK
P76342	protein-L-methionine sulfoxide reductase catalytic subunit	*msrP*	0.3	1.9	2.6	KG
P0ADU2	putative quinol monooxygenase	*ygiN*	1.92	2.4	0.3	TKG
P42593	2,4-dienoyl-CoA reductase	*fadH*	1.49	2.6	3.6	K
P11868	propionate kinase	*tdcD*	1.24	2.1	0.8	TK
P06715	glutathione reductase (NADPH)	*gor*	1.33	4.7	0.7	TK
P32662	phosphoglycolate phosphatase	*gph*	0.4	5.2	0.8	N
POABK2	cytochrome bd-I subunit 2	*cydB*	0.79	30.2	3	TKG
P23304	putative quinone oxidoreductase 1	*qorA*	0.61	4.2	5	TKG
**E, Amino acid transport and metabolism**						
P00909	fused indole-3-glycerol phosphate synthase/phosphoribosylanthranilate isomerase	*trpC*	1.55	3.9	3	TKG
P76038	gamma-glutamyl-gamma-aminobutyrate hydrolase	*puuD*	1.97	4.2	1.0	N
P37906	gamma-glutamylputrescine oxidase	*puuB*	1.64	2.1	1.6	KG
P50457	4-aminobutyrate aminotransferase	*puuE*	0.95	4.3	2.4	N
P76043	D-guloside 3-dehydrogenase	*ycjQ*	2.3	5.6	4.4	N
P77795	putative ABC transporter ATP-binding protein	*ycdT*	0.59	5.5	1.2	KG
P05194	3-dehydroquinate dehydratase	*aroD*	0	5.9	1.7	TK
P0AE37	arginine N-succinyltransferase	*astA*	2.03	3.3	0.23	KG
P77539	putative zinc-binding dehydrogenase	*ydjL*	3.63	3.9	2	N
P77529	cystine/sulfocysteine:cation symporter	*tcyP*	0.22	26.9	1.6	N
P76316	D-cysteine desulfhydrase	*dcyD*	0.91	2.8	3.6	TKG
P60595	imidazole glycerol phosphate synthase subunit	*hisH*	2.04	6.5	5.2	TKG
P22256	4-aminobutyrate aminotransferase	*gabT*	1.88	4.1	2.3	TK
P06721	cystathionine beta-lyase/L-cysteine	*metC*	1.52	3.9	2.1	TKG
P42588	putrescine aminotransferase	*patA*	1.96	4.1	2.6	N
P0AAG0	dipeptide ABC transporter ATP binding subunit	*dppD*	1.83	6.3	2.7	TK
P23847	dipeptide ABC transporter periplasmic binding protein	*dppA*	0.75	4	1.1	TKG
P0AAE0	D-serine/alanine/glycine/:H(+)symporter	*cyrA*	1.28	33.5	2.2	N
P39346	L-idonate 5-dehydrogenase	*idnN*	2.92	1.7	1.1	K
**F, Nucleotide transport and metabolism**						
P15723	dGTP triphosphohydrolase	*dgt*	1.2	2.2	1.2	TKG
P0AF24	UMP phosphatase	*nagD*	1.2	3.6	0.9	G
P0A720	Thymidylate kinase	*tmk*	0	4.6	0.1	TKG

P0ACE7	purine nucleoside phosphoramidase	*hinT*	0	0.8	1.1	TK
P31806	NAD(P)HX epimerase/NAD(P)HX dehydratase	*nnr*	1.17	2.9	2.8	N
**G, Carbohydrate transport and metabolism**						
P36881	putative PTS enzyme IIA component	*yadL*	3.42	4.1	0.6	G
P77364	glycerate 2-kinase 2	*glxK*	2.36	3.7	2.0	N
P52697	6-phosphogluconolactonase	*pgl*	1.51	5.9	3.1	N
P13482	periplasmic trehalase	*treA*	0.7	2.8	2.2	N
P77432	autoinducer-2 kinase	*isrK*	1.89	2.5	1.7	KG
P76142	Autoinducer ABC transporter periplasmic binding protein	*isrB*	0.59	7.2	5.3	N
P77739	putative kinase	*yniA*	1.4	2	2.4	G
P02924	arabinose ABC transporter periplasmic binding	*araF*	0.3	4.2	2.03	S
P69795	N,N-diacetylchitobiose-specific PTS enzyme IIB component	*chbB*	0.94	1.33	2	TK
P26612	alpha-amylase	*amyA*	1.41	5.1	2.1	TKG
P77272	N-acetylmuramic acid-specific PTS enzyme IICB component/anhydro-N-acetylmuramic acid transporter	*murP*	1.48	22.3	1.3	N
P33570	transketolase 2	*tktB*	0.6	3.7	2.3	TK
P0AE24	arabinose:H(+) symporter	*araE*	1.06	27.6	1.6	KG
P42904	N-acetyl-D-galactosamine specific PTS enzyme IIB component	*agaV*	1.27	9	0.2	N
P37388	xylose ABC transporter ATP binding subunit	*xylG*	1.95	2.9	3.03	K
P37691	divergent polysaccharide deacetylase domain-containing protein	*yibQ*	0.31	6.6	2.1	KG
P02925	ribose ABC transporter periplasmic binding protein	*rbsB*	0	7.2	2	TKG
P0C0L7	osmolyte:H(+) symporte	*proP*	0.8	27.6	3.02	S
P39325	galactofuranose ABC transporter periplasmic binding protein	*ytfQ*	0.63	2.8	0.6	N
P32670	putative PTS multiphosphoryl transfer protein	*ptsA*	2.04	2.6	2.9	N
**H, Coenzyme transport and metabolism**						
P0AG40	bifunctional riboflavin kinase/FMN adenylyltransferase	*ribF*	1.28	3.1	1.7	TKG
P26281	2-amino-4-hydroxy-6-hydroxymethyldihydropteridine diphosphokinase	*folK*	0	4.3	0.4	G
P07821	iron(III) hydroxamate ABC transporter ATP binding subunit	*fhuC*	0.75	7.3	0.1	N
P60720	lipoyl(octanoyl) transferase	*lipB*	1.41	4.6	2.5	TK
P06611	vitamin B12 ABC transporter ATP binding subunit	*btuD*	0.4	3.2	0.14	KG
P0A8Y3	alpha-D-glucose-1-phosphate phosphatase	*yihX*	1.01	7.1	1.4	TKG
P06983	hydroxymethylbilane synthase	*hemC*	1.28	2.7	1.7	KG
**I, Lipid transport and metabolism**						
P45568	1-deoxy-D-xylulose 5-phosphate reductoisomerase	*dxr*	2.01	4.9	1.3	G

Q47146	acyl-CoA dehydrogenase	*fadE*	0.98	8.5	2.2	TK
P62615	4-(cytidine 5-diphospho)-2-C-methyl-D-erythritol kinase	*ispE*	1.41	6.1	2.3	TK
P0A9P9	5-keto-D-gluconate 5-reductase	*idnO*	1.18	8	2.7	TK
P76149	succinate semialdehyde dehydrogenase (NAD(P)(+))	*sad*	1.52	3.1	2.2	K
**P, Inorganic ion transport and metabolism**						
P03819	K(+) : H(+) antiporter	*kefC*	0.65	18.4	2.8	N
P0AAC4	Bax1-I family protein	*ybhL*	0	42.6	0.1	N
P16869	ferric coprogen/ferric rhodotorulic acid outer membrane transporter	*fhuE*	0.14	1.23	1.31	S
P37327	inner membrane protein	*yfdC*	0.32	19.4	2.6	N
P0ADE6	(+) binding protein	*ygaU*	0	4.5	8.5	N
P0A6J1	adenylyl-sulfate kinase	*cysC*	1	7.4	0.7	N
P0AG82	phosphate ABC transporter periplasmic binding protein	*pstS*	0	11.2	2.2	TG
P37908	UPF0053 family inner membrane protein	*yfjD*	0	10.9	3.4	N
**J, Translation, ribosomal structure and biogenesis**						
P0AFW2	ribosome modulation factor	*rmf*	1.82	0.01	0	N
P13857	ribosomal-protein-L12-serine N-acetyltransferase	*rimL*	1.68	4.2	4.1	G
P0A7Q1	50S ribosomal subunit protein L35	*rpmL*	1.54	2.1	0.01	N
P65581	ribosome- and membrane-associated DUF883 domain-containing protein	*yqjD*	0	10.5	0	KG
P0AM4	D-aminoacyl-tRNA deacylase	*dtd*	1.38	10.8	1.7	N
P0A8N7	EF-P-lysine lysyltransferase	*empA*	1.54	5.2	2.2	TKG
**K, Transcription**						
P0AAP3	DNA-binding transcriptional repressor	*frmR*	2.2	0.2	0.02	N
POABE2	DNA-binding transcriptional dual regulator	*bolA*	0.95	3	0.03	K
P0A8B5	putative nucleoid-associated protein	*ybaB*	0.92	0.2	0.5	TKG
P0AFM6	phage shock protein A	*pspA*	0	0.1	0.2	TK
P0ACS5	DNA-binding transcriptional activator	*zntR*	3.55	0.2	0.4	S
P0A8U6	DNA-binding transcriptional repressor	*metJ*	0.95	0.05	0.08	S
P09163	peptidyl-lysine N-acetyltransferase	*yjaB*	1.36	7.02	0.5	N
P0A9V5	putative DNA-binding transcriptional regulator	*yiaG*	1.04	1.04	5.1	TKG
P16691	aminoalkylphosphonate N-acetyltransferase	*phnO*	1.4	2.2	1.7	K
P31460	DNA-binding transcriptional regulator	*dgoR*	0.44	2.5	2.7	TK
**L, Replication, recombination and repair**						
P0A8N0	macrodomain Ter protein	*matP*	0	1.8	0.8	KG
P0DM85	regulator of diguanylate cyclase RdcA	*crfC*	1.21	2.7	2.8	TK
P15943	ATP-dependent DNA helicase	*recQ*	1.81	5.02	1.6	TK
P0ADX9	16S rRNA m(2)G966 methyltransferase	*rsmD*	1.01	1.3	4.3	N
**M, Cell wall/membrane/envelope biogenesis**						
P13016	1,6-anhydro-N-acetylmuramoyl-L-alanine amidase	*ampD*	2.73	1.9	0.5	N
P23930	apolipoprotein N-acyltransferase	*Int*	0.59	16	1.8	N
P75785	phosphoethanolamine transferase	*opgE*	0.8	15.3	4	G
P22525	L,D-transpeptidase LdtD	*ycbB*	0.65	3.4	3.8	N

P0AB38	outer membrane lipoprotein - activator of MrcB activity	*lpoB*	0.47	6.2	2.8	K
P02929	Ton complex subunit TonB	*tonB*	0.42	8.4	0.7	K
P76206	acid-inducible putative outer membrane protein	*ydiY*	0	2.2	1.7	N
P0ADV9	lipopolysaccharide transport system protein	*lptC*	0	10.7	4.7	N
P37645	AsmA family protein	*yhjG*	0.29	5.1	1.6	N
P27836	UDP-N-acetyl-D-mannosaminuronic acid transferase	*wecG*	0.41	2.8	9.8	K
P25742	lipopolysaccharide core heptosyltransferase 3	*rfaQ*	1.74	5.3	1.6	G
P0A901	outer membrane lipoprotein	*blc*	1.13	11.4	1.7	TK
P0ADE4	translocation and assembly module subunit	*tamA*	0.52	1.9	3.8	G
P0AF98	lipopolysaccharide transport system protein	*lptF*	0.55	22.8	0.9	N
P0ADC6	lipopolysaccharide transport system protein	*lptG*	0	18.4	5.1	N
P0AFH8	periplasmic chaperone	*osmY*	0	6.1	0.04	TK
**O, Post translational modification, protein turnover, chaperones**						
P31680	co-chaperone protein	*djlA*	0.74	9.8	1.2	N
P0A6G7	ATP-dependent Clp protease proteolytic subunit	*clpP*	0.97	9.2	2.8	TKG
P75796	glutathione ABC transporter ATP binding subunit	*gsiA*	0.64	5.6	2.5	KG
P75867	putative ATP-dependent protease YcbZ - Lon	*ycbZ*	1.19	3.4	4.5	TK
P0A6L9	[Fe-S] cluster biosynthesis co-chaperone	*hscB*	0	2.1	0.03	N
P0AAN3	hydrogenase maturation factor HypB	*hypB*	3.1	4	1.6	K
P0AEG6	protein disulfide isomerase	*dsbC*	1.69	7.7	1.6	TK
**Q, Secondary metabolites biosynthesis, transport and catabolism**						
P36566	tRNA cmo(5)U34 methyltransferase	*cmoM*	1.53	5.1	0.2	KG
**T, Signal transduction mechanisms**						
P0AEV1	regulator of RpoS	*rssB*	1.78	4.5	2.9	TK
P76046	DUF463 domain-containing protein	*ycjX*	0.86	1.6	3.1	TKG
P07018	methyl-accepting chemotaxis protein	*tap*	0.19	7.9	1	N
P50466	aerotaxis receptor	*aer*	0.59	7.1	1.6	G
P69829	phosphotransferase system enzyme IIA(Ntr)	*ptsN*	1.84	6.2	7.3	TK
P0A894	RNase adaptor protein RapZ	*rapZ*	0.7	4.1	2.1	TKG
P13518	regulator of CsrB and CsrC decay	*csrD*	0.77	6.8	0.4	N
P37649	c-di-GMP phosphodiesterase	*pdeK*	1.06	11.1	2.5	N
**V, Defense mechanisms**						
P37636	multidrug efflux pump membrane fusion protein	*mdtE*	0.52	2.6	0.9	KG
**U, Intracellular trafficking, secretion, and vesicuar transport**						
P0ABV6	Tol-Pal system protein	*tolR*	0	12.8	1.9	T
P0AG96	Sec translocon subunit	*secE*	0	34.6	1.9	K
**POORLY CHARACTERIED**						
**R, General function prediction only**						

Q47147	putative glutamine	*yafJ*	2.75	6	1.3	TKG
P76235	DUF444 domain-containing protein	*yeaH*	0.47	3.0	4.9	N
P52007	putative metal-binding enzyme	*yecM*	1.6	0.5	2.1	K
P77148	FAD/NAD(P) binding domain-containing protein	*ydhS*	0.94	3.2	3.1	N
P42599	putative oxidoreductase	*yqjR*	2.13	3.9	5.2	TKG
P0ADK6	putative lyase containing HEAT-repeat	*yibA*	2.86	6.1	5.2	TK
P31469	PF03691 family colicin E2 tolerance protein	*cbrC*	6.67	3.8	2.7	N
P39315	NAD(P)H:quinone oxidoreductase	*qorB*	0	5.2	4.3	N
**S, Function unknown**						
P0ADA5	putative lipoprotein	*yajG*	0.52	7.4	3.8	K
P0A8R7	DUF697 domain-containing inner membrane protein	*ycjF*	0.85	7.5	3.1	TKG
P76231	DUF1315 domain-containing protein	*yeaC*	1.1	3.0	4.9	S
P33355	DUF1456 domain-containing protein	*yehS*	1.28	6.6	0.5	N
P64585	inner membrane protein	*yqjE*	0	23.5	0.01	N
**Non categories**						
P69411	sensor lipoprotein	*rcsF*	4.48	4.9	0.4	KG
P77562	surface-exposed outer membrane lipoprotein	*yaiW*	0.82	3.8	2.1	N
P0AB03	putative phosphotransferase	*ycbJ*	1.68	4.6	1.3	G
P0ADB1	osmotically inducible lipoprotein	*osmE*	2.68	8.4	0.5	N
P31063	lipoprotein	*yedD*	2.19	6.5	0.9	N
Q47710	PF13997 family protein	*yqjK*	0	6.2	1.4	N
P0ADX7	DUF2756 domain-containing protein	*yhhA*	0	3.1	0.01	N
P0AF70	DUF4156 domain-containing lipoprotein	*yjeI*	2.56	6.2	3.02	TKG

*normalized by protein length.

** Chaperone client classification: **G**, GroEl/ES; **N**, proteins showing no chaperone dependence in any of the experiments; **K**, DnaK/J; **S**, contradictory results (i.e. increased mRNA or protein abundance upon chaperone deletion, and no other apparent chaperone dependencies in any of the studies); **T**, Trigger Factor. (Ramakrishnan et al., 2019).

Protein Homeostatis Database (http://phdb.switchlab.org/#/home)
