# Supplementary figures and images for: The Thioredoxin Fold Protein (TFP2) from Extreme Acidophilic Leptospirillum sp. CF-1 Is a Chaperedoxin-like Protein That Prevents the Aggregation of Proteins under Oxidative Stress
Source: Int J Mol Sci. 2024 Jun 24;25(13):6905. doi: 10.3390/ijms25136905 (PMC11241051; doi:10.3390/ijms25136905)

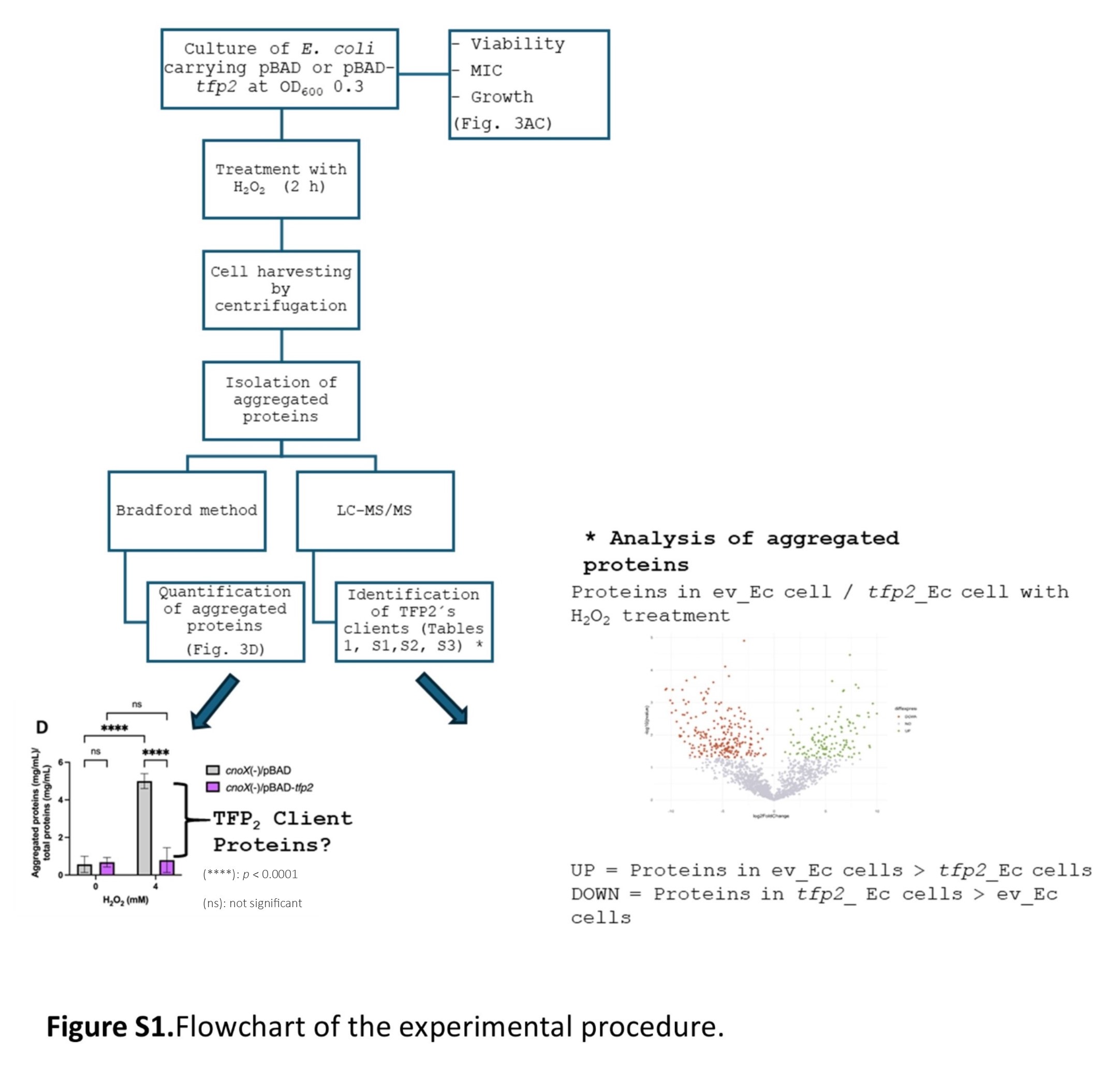

Supplement: Supplementary file 1 [file ijms-25-06905-s001.zip › Figure S1.jpg]
